# Candida dubliniensis fungemia: the first four cases in North America.

**DOI:** 10.3201/eid0601.000108

**Published:** 2000

**Authors:** M E Brandt, L H Harrison, M Pass, A N Sofair, S Huie, R K Li, C J Morrison, D W Warnock, R A Hajjeh

**Affiliations:** Centers for Disease Control and Prevention (CDC) Atlanta, Georgia, USA. mbb4@cdc.gov

## Abstract

We report the first four North American cases of Candida dubliniensis fungemia, including the first isolation of this organism from the bloodstream of an HIV-infected person. All isolates were susceptible in vitro to commonly used antifungal drugs. This report demonstrates that C. dubliniensis can cause bloodstream infection; however, the incidence of disease is not known.


### *Candida dubliniensis* Fungemia: the First Four Cases in North America

**Mary E. Brandt,* Lee H. Harrison,†‡ Margaret Pass,† Andre N. Sofair,§ Sharon Huie,§ Ren-Kai Li,* Christine J. Morrison,* David W. Warnock,* and Rana A. Hajjeh*[1]**

*Centers for Disease Control and Prevention (CDC), Atlanta, Georgia, USA; †Johns Hopkins University School of Hygiene and Public Health, Baltimore, Maryland, USA; ‡University of Pittsburgh Graduate School of Public Health, Pittsburgh, Pennsylvania, USA; and §Yale University School of Medicine, New Haven, Connecticut, USA.

We report the first four North American cases of *Candida dubliniensis* fungemia, including the first isolation of this organism from the bloodstream of an HIV-infected person. All isolates were susceptible in vitro to commonly used antifungal drugs. This report demonstrates that *C. dubliniensis* can cause bloodstream infection; however, the incidence of disease is not known.

In recent years, *Candida* species other than *C. albicans* have emerged as causes of human candidiasis, particularly in HIV-infected and other immunocompromised persons (1). *C. dubliniensis,* a recently described species closely related to *C. albicans* (2), has been implicated as an agent of oral candidiasis in HIV-positive persons (2-5) but has also been recovered from HIV-negative persons with clinical signs of oral candidiasis and from the genital tract of some women with vaginitis (2,4). First isolated from AIDS patients in Dublin, Ireland (2), *C. dubliniensis* has a worldwide distribution (3-6). Most isolates are susceptible to amphotericin B and the azoles, but resistance has been shown in HIV-positive patients on fluconazole for oral candidiasis (7). Its potential to cause deep or disseminated candidiasis is not known, largely because *C. dubliniensis* has rarely been isolated from sterile body sites (6); however, the phenotypic characteristics the organism shares with *C. albicans* (producing germ tubes and chlamydospores) suggest that some *C. dubliniensis* isolates may have been misidentified as *C. albicans*.

Address for correspondence: Mary E. Brandt, Mycotic Diseases Branch, Centers for Disease Control and Prevention, 1600 Clifton Road, Mailstop G11, Atlanta, GA 30333, USA; fax: 404-639-3546; e-mail: mbb4@cdc.gov.

Three cases of *C. dubliniensis* fungemia have been reported from Europe, all in HIV-negative bone marrow transplant recipients with chemotherapy-induced neutropenia (8). We report the first isolation of *C. dubliniensis* from the blood cultures of four patients from the United States. All four patients had multiple underlying conditions and at least one symptom of septicemia (fever, hypotension, or multiple organ system failure) at the time blood cultures were drawn. These cultures were collected from October 1998 to January 1999 through active, population-based laboratory surveillance for candidemia in residents of Connecticut and of Baltimore City or County, Maryland (combined population, 4.8 million). An incident case of candidemia was defined by the first isolation of any *Candida* species from a blood culture from a resident of one of the surveillance areas. Medical records were reviewed to obtain demographic data, clinical data on underlying medical conditions, treatment, and outcome.

### Case 1

A 74-year-old black man from Baltimore, with a history of chronic lymphocytic leukemia, chronic obstructive pulmonary disease, coronary artery disease and hypertension, was hospitalized for fatigue and anemia 4 weeks after

[1]For the CDC Candidemia Surveillance Group, which includes W. Ruth Pruitt, Nathelia LeSane, Gabriel Ponce de Leon, Meral Ciblak, G. Marshall Lyon (CDC); Laurie Thomson Sanza (Maryland); Lily Yeo and Brian Wong (Connecticut).

chemotherapy with chlorambucil. He received multiple blood transfusions on day 1 of hospitalization and quickly progressed to multiple organ failure, including renal failure and hepatic and cardiogenic shock, and was transferred to a medical intensive care unit. Multiple indwelling catheters were placed. A peripheral blood culture obtained 3 days after admission grew C. *dubliniensis*. The patient died 1 day after this single positive blood culture was drawn. No autopsy was performed.

### Case 2

A 30-year-old black woman from Connecticut with a history of end-stage liver disease was hospitalized for gastrointestinal bleeding and refractory ascites. The patient had a history of intravenous drug use and alcoholism but was not infected with HIV. During hospitalization, she required multiple transfusions; acute renal failure requiring hemodialysis followed. The patient was receiving many medications, including vasopressors, antibiotics, and corticosteroids. Yeasts were isolated from peripheral blood cultures collected on days 11, 15, and 17 of hospitalization; four of the isolates were later identified as *C. dubliniensis*. The patient had a triple-lumen catheter placed on the day before the initial isolation of the organism. She was treated with intravenous fluconazole 200 mg/day for 5 days, starting 6 days after the first yeast isolation. A blood culture collected on day 20 was negative, and the patient died after 24 days of hospitalization. No autopsy was performed.

### Case 3

The third patient was a 39-year-old black man from Baltimore who was admitted for complications of end-stage liver disease, including acute renal failure and ascites, and diffuse lymphadenopathy of unknown etiology. He also had a history of diabetes mellitus. A week before hospitalization, the patient had been discharged from another hospital, where he had been admitted because of pancreatitis and treated for *Escherichia coli* bacteremia and renal insufficiency. The patient had a peripheral intravenous catheter and a central venous catheter placed during this hospitalization. On day 2, yeasts were recovered from a peripheral blood culture; these were later identified as *C. dubliniensis*. By the time the result of the blood culture was reported, the patient's clinical status had deteriorated because of worsening respiratory distress. He was treated with fluconazole 400 mg/day for 3 days but died 5 days after the blood culture was obtained. No autopsy was performed.

### Case 4

The fourth case occurred in a 37-year-old white woman from Baltimore, who had a history of intravenous drug use, chronic deep vein thrombosis, and valvular heart disease. She was also HIV-infected ($CD4^+$ lymphocyte count was 779 cells/ml). She was hospitalized because of fever and chills, and blood cultures on day 1 of admission grew group A streptococci, for which she was treated with various antibiotics. On day 7, fever developed and peripheral blood cultures grew *C. dubliniensis* and *C. glabrata*. She was treated with oral fluconazole 400 mg/day for 2 weeks and was discharged to a skilled-nursing facility 1 day after being started on fluconazole.

### Microbiologic Results

All seven isolates were originally identified as *C. albicans* on the basis of their phenotypic characteristics. They were reexamined at the Fungus Reference Laboratory, Centers for Disease Control and Prevention, and reidentified as *C. dubliniensis* on the basis of biochemical and morphologic criteria (9). The identification was confirmed by reactivity of DNA with a polymerase chain reaction (PCR)-enzyme immunoassay (EIA) probe specific for this species (10) and by PCR amplification of a region containing the novel *C. dubliniensis* group I intron in the large ribosomal subunit (11).

Broth microdilution MICs were determined according to National Committee for Clinical Laboratory Standards document M27-A guidelines (12). All isolates were susceptible to commonly used antifungal agents. MICs of amphotericin B were 0.25 (one patient) to 0.5 µg/ml (three patients); MICs of itraconazole were from <0.015 (two patients) to 0.03 µg/ml (two patients): and MICs of fluconazole and flucytosine were <0.125 µg/ml for all isolates.

### Conclusions

The incidence of candidemia due to *C. dubliniensis* is not known, largely because of the difficulty in readily distinguishing this species from the morphologically similar *C. albicans*. However, in laboratory-based surveillance conducted in 1992-93 in two sites in the United States (population 5.8 million), we did not find *C. dubliniensis* as an agent of candidemia, even with the DNA-based identification method used in this study (13). More recently, three cases of *C. dubliniensis* fungemia have been reported from Europe in patients with chemotherapy-induced immunosuppression and bone marrow transplantation (8). The four cases described here are the first reported in the United States.

The demonstration that *C. dubliniensis* has the potential to cause bloodstream infection provides information central to our understanding of its clinical relevance and pathogenic potential. As in the earlier European report (8), the patients in our study had multiple serious medical conditions. Two of the four patients had end-stage liver disease, which is a known risk factor for bloodstream infections with organisms that are part of the normal gastrointestinal flora because of breakdown of the normal mucosal barrier (14). This strongly suggests that the gastrointestinal tract was the source of the *C. dubliniensis* in these patients. Odds et al. (6) have reported the reidentification as *C. dubliniensis* of a number of *C. albicans* isolates that were obtained from fecal surveillance cultures in hematologic patients.

The isolation of *C. dubliniensis* from mucosal sites in HIV-infected persons has been widely reported (3,5). Although not severely immunocompromised, our fourth patient was HIV-positive, which makes hers the first reported case of bloodstream infection with this organism in an HIV-infected person. The fact that *C. dubliniensis* is able to cause invasive disease in these patients is of clinical interest. However, it may be more significant that *C. glabrata*, a recognized pathogen, was also isolated from blood cultures in our patient.

As our population-based surveillance for candidemia continues, we will be able to estimate more accurately the incidence of candidemia due to *C. dubliniensis* and define more clearly its clinical importance, epidemiologic characteristics, and outcome. The specific proportional impact of *C. dubliniensis* candidemia on outcome is difficult to assess in these patients, all of whom had multiple underlying conditions. The organisms isolated from our patients were all fully susceptible to amphotericin B, flucytosine, fluconazole, and itraconazole. However, resistance has been shown to occur in HIV-positive patients given fluconazole treatment for oral infection with *C. dubliniensis* (7). As our knowledge about this emerging pathogenic yeast increases and diagnostic tests are developed, prevention and better management of the disease will become possible.

### Acknowledgment

We thank the laboratory participants who collected isolates for this study.

Dr. Brandt is a research microbiologist in the Mycotic Diseases Branch, Division of Bacterial and Mycotic Diseases, CDC. She and Dr. Hajjeh are members of the CDC Fungal Active Surveillance Group, which conducts active population-based surveillance for fungal diseases of public health importance.

### References

1. Coleman DC, Rinaldi MG, Haynes KA, Rex JH, Summerbell RC, Anaissie EJ, et al. Importance of *Candida* species other than *Candida albicans* as opportunistic pathogens. Medical Mycology 1998;36 Suppl 1:156-65.
2. Sullivan DJ, Westerneng TJ, Haynes KA, Bennett DE, Coleman DC. *Candida dubliniensis* sp. nov.: phenotypic and molecular characterization of a novel species associated with oral candidosis in HIV-infected individuals. Microbiology 1995;141:1507-21.
3. Coleman DC, Sullivan DJ, Bennett DE, Moran GP, Barry HJ, Shanley DB. Candidiasis: the emergence of a novel species, *Candida dubliniensis*. AIDS 1997;11:557-67.
4. Sullivan D, Coleman D. *Candida dubliniensis*: characteristics and identification. J Clin Microbiol 1998;36:329-34.
5. Sullivan D, Haynes K, Bille J, Boerlin P, Rodero L, Lloyd S, et al. Widespread geographic distribution of oral *Candida dubliniensis* strains in human immunodeficiency virus-infected individuals. J Clin Microbiol 1997;35:960-4.
6. Odds FC, Van Nuffel L, Dams G. Prevalence of *Candida dubliniensis* isolates in a yeast stock culture collection. J Clin Microbiol 1998;36:2869-73.
7. Moran GP, Sullivan DJ, Henman MC, McCreary CE, Harrington BJ, Shanley DB, et al. Antifungal drug susceptibilities of oral *Candida dubliniensis* isolates from human immunodeficiency virus (HIV)-infected and non-HIV-infected subjects and generation of stable fluconazole-resistant derivatives in vitro. Antimicrob Agents Chemother 1997;41:616-23.
8. Meis JFGM, Ruhnke M, DePauw BE, Odds FC, Siegert W, Verweij PE. *Candida dubliniensis* candidemia in patients with chemotherapy-induced neutropenia and bone marrow transplantation. Emerg Infect Dis 1999;5:150-3.
9. Salkin IF, Pruitt WR, Padhye AA, Sullivan D, Coleman D, Pincus DH. Distinctive carbohydrate assimilation profiles used to identify the first clinical isolates of *Candida dubliniensis* recovered in the United States. J Clin Microbiol 1998;36:1467.

10. Elie CM, Lott TJ, Reiss E, Morrison CJ. Rapid identification of *Candida* species with species-specific DNA probes. J Clin Microbiol 1998;36:3260-5.
11. Boucher H, Mercure S, Montplaisir S, Lemay G. A novel group I intron in *Candida dubliniensis* is homologous to a *Candida albicans* intron. Gene 1996;180:189-96.
12. National Committee for Clinical Laboratory Standards. Reference method for broth dilution antifungal susceptibility testing of yeasts. Approved Standard M27-A. Wayne (PA): The Committee;1997.
13. Elie CM, Kao AS, Hajjeh RA, Brandt ME, Pruitt WR, Reiss E, et al. Examination of bloodstream yeast isolates for the presence of the recently described *Candida* species, *C. dubliniensis*, as part of a prospective, population-based surveillance study. In: Abstracts of the Fifth Candida and Candidiasis Conference, 1999. Abstract C41, p. 60. Washington: American Society for Microbiology.
14. Cole GT, Halawa AL, Anaissie EJ. The role of the gastrointestinal tract in hematogenous candidiasis: from the laboratory to the bedside. Clin Infect Dis 1996;22 Suppl 2:S73-88.
